# Mechanisms of Osteoarthritic Pain. Studies in Humans and Experimental Models

**DOI:** 10.3389/fnmol.2017.00349

**Published:** 2017-11-03

**Authors:** Annett Eitner, Gunther O. Hofmann, Hans-Georg Schaible

**Affiliations:** ^1^Department of Physiology, University Hospital Jena, Friedrich Schiller University, Jena, Germany; ^2^Department of Traumatology and Orthopedic Surgery, University Hospital Jena, Friedrich Schiller University, Jena, Germany; ^3^Trauma Center Bergmannstrost Halle, Halle, Germany

**Keywords:** osteoarthritis, pain mechanisms, synovitis, cytokines, nerve growth factor, diabetes mellitus, neuropathic pain, pain treatment

## Abstract

Pain due to osteoarthritis (OA) is one of the most frequent causes of chronic pain. However, the mechanisms of OA pain are poorly understood. This review addresses the mechanisms which are thought to be involved in OA pain, derived from studies on pain mechanisms in humans and in experimental models of OA. Three areas will be considered, namely local processes in the joint associated with OA pain, neuronal mechanisms involved in OA pain, and general factors which influence OA pain. Except the cartilage all structures of the joints are innervated by nociceptors. Although the hallmark of OA is the degradation of the cartilage, OA joints show multiple structural alterations of cartilage, bone and synovial tissue. In particular synovitis and bone marrow lesions have been proposed to determine OA pain whereas the contribution of the other pathologies to pain generation has been studied less. Concerning the peripheral neuronal mechanisms of OA pain, peripheral nociceptive sensitization was shown, and neuropathic mechanisms may be involved at some stages. Structural changes of joint innervation such as local loss and/or sprouting of nerve fibers were shown. In addition, central sensitization, reduction of descending inhibition, descending excitation and cortical atrophies were observed in OA. The combination of different neuronal mechanisms may define the particular pain phenotype in an OA patient. Among mediators involved in OA pain, nerve growth factor (NGF) is in the focus because antibodies against NGF significantly reduce OA pain. Several studies show that neutralization of interleukin-1β and TNF may reduce OA pain. Many patients with OA exhibit comorbidities such as obesity, low grade systemic inflammation and diabetes mellitus. These comorbidities can significantly influence the course of OA, and pain research just began to study the significance of such factors in pain generation. In addition, psychologic and socioeconomic factors may aggravate OA pain, and in some cases genetic factors influencing OA pain were found. Considering the local factors in the joint, the neuronal processes and the comorbidities, a better definition of OA pain phenotypes may become possible. Studies are under way in order to improve OA and OA pain monitoring.

## Introduction

Pain due to osteoarthritis (OA) is one of the most frequent causes of chronic pain (Breivik et al., 2006). The significance of OA pain is thought to increase in an aging population. Knees, hips and hands are the most commonly affected joints. For example, as reviewed by Neogi (2013) the prevalence of radiographic knee OA was 19% and 28% among adults age >45 years in the Framingham study and in the Johnston County OA Project, respectively, while the prevalence of symptomatic knee OA (showing symptoms in addition to radiographic evidence) was 7% in the Framingham study and 17% in the Johnston County OA Project. The prevalence of symptomatic knee OA in two UK studies ranged from 11% to 19%, and estimates of 5%–15% were noted in surveys undertaken in other countries (Neogi, 2013). Interestingly, symptomatic hand OA is far less common. Several studies reported a prevalence of only 6%–8% in the general population (Carmona et al., 2001; Dillon et al., 2007), and 13%–26% in the elderly population (Zhang et al., 2002). Often several joints of the hand are affected (Zhang et al., 2002; Marshall et al., 2013). OA may also appear as polyarticular OA and is then called “generalized OA”. However, because there is no clear definition of “generalized OA”, it is difficult to estimate its prevalence and risk factors (Nelson et al., 2014).

This review addresses the mechanisms which are thought to be involved in OA pain. Three levels have to be considered, namely: (i) the local pathological processes in the OA joint leading to OA pain; (ii) the neuronal mechanisms and alterations of pain processing which are involved in OA pain; and (iii) general factors such as genetic and metabolic factors which may play a role in OA pain. The review will refer to human OA as well as to experimental OA models. It has to be noted that the vast majority of the pain studies has been made for knee OA. It should be kept in mind that there might be differences in OA of other joints.

## Local Processes in the Joint Related to Pain

### Human OA

While the hallmark of OA is the progressive destruction of cartilage, OA is now considered a disease of the whole joint (Felson, 2009; Hofmann et al., 2010; Goldring and Otero, 2011; Yusup et al., 2015). The subchondral bone may show sclerosis, combined with osteophytes (Burr and Gallant, 2012). Furthermore, OA is often associated with inflammatory changes in the joint such as synovitis (Goldring and Otero, 2011; Attur et al., 2015; Berenbaum and van den Berg, 2015; Yusup et al., 2015). Furthermore, OA joints may show bone marrow lesions (Zhang et al., 2011).

**Figure 1:**
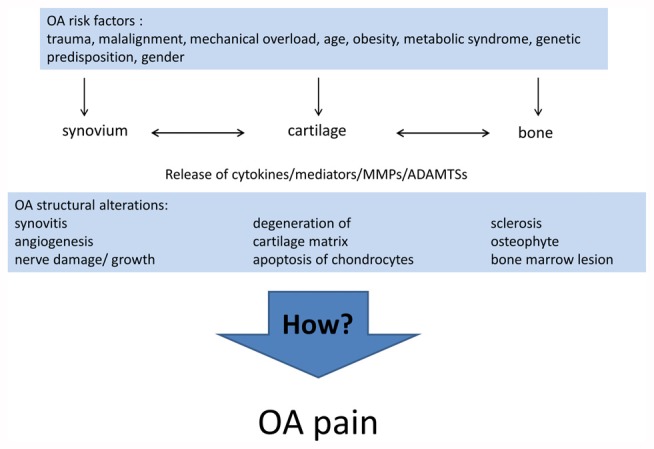
Risk factors and pathological events leading to osteoarthritis (OA).

Differences in the individual phenotype of OA may result from the different causes and pathogenic processes leading to OA. Currently, the list of risk factors for the development of OA includes joint injury (Gelber et al., 2000; Blagojevic et al., 2010), malalignment and other mechanical factors (Gelber et al., 1999; Sharma et al., 2001; Felson, 2009; Hofmann et al., 2010; Toivanen et al., 2010; Felson et al., 2013), age (Blagojevic et al., 2010), gender (Srikanth et al., 2005), obesity (Grotle et al., 2008; Blagojevic et al., 2010; Berenbaum et al., 2017), the metabolic syndrome (Yoshimura et al., 2012; Berenbaum and van den Berg, 2015; Courties et al., 2015; Wang et al., 2015; Berenbaum et al., 2017), in particular diabetes mellitus (Schett et al., 2013; Eymard et al., 2015; King and Rosenthal, 2015), and genetic predisposition (Spector et al., 1996; Loughlin, 2005). Inflammation *per se* is now considered a risk factor for OA progression (Larsson et al., 2015; Lieberthal et al., 2015). For early stages of OA Siebuhr et al. (2016) described four subpopulations of OA depending on the main driver of disease progression: synovium-driven OA (characterized by inflammation), cartilage-driven OA, OA driven by the subchondral bone and bone marrow lesions, OA driven by trauma, meniscus lesion and others. At advanced stages of OA different pathological processes may be combined and lead to a similar end stage phenotype. Figure 1 summarizes the risk factors and structural alterations of OA development.

The heterogeneity of pathological changes raises the question whether particular structural and pathogenic changes can be identified which are linked to pain. Often, a poor relationship between radiographic images and pain was reported. A systematic literature search of Bedson and Croft (2008) showed that 15%–76% of the patients with knee pain had radiographic indications of OA, strongly depending on the study design concerning applied technics and scorings of structural changes and clinical symptoms. The prevalence of knee pain in patients with radiographic knee OA ranged from 15% to 81% (Bedson and Croft, 2008). However, some studies reported associations between the structural damage of the joint (cartilage and bone) and pain (Malfait and Schnitzer, 2013). E.g., knee pain occurred in a higher proportion of OA patients with Kellgren/Lawrence (K/L) grade 4 than of OA patients with K/L grades 2 and 3 (Neogi et al., 2009). In a longitudinal study, knees with frequent pain displayed greater rates of medial cartilage loss (also after adjustment for radiographic OA stage; Eckstein et al., 2011). Osteophytes were strongly associated with knee pain (Kaukinen et al., 2016). In interphalangeal joint OA, patients with erosive OA showed more pain and functional impairment than patients with non-erosive OA (Wittoek et al., 2012). Thus pain may indicate the disease activity.

Recent research focused on associations of pain with pathological changes which are particularly visible in MRI images. Zhang et al. (2011) for example reported that pain in knee OA fluctuates with changes of bone marrow lesions and synovitis. When bone marrow lesions become smaller, the pain is reduced, and the risk of frequent pain decreases. By contrast, worsening of synovitis and effusions are associated with increased risk of frequent and more severe pain (Zhang et al., 2011). A positive relationship between inflammatory changes in the joint and pain was also shown in recent MRI studies (de Lange-Brokaar et al., 2015; Yusup et al., 2015; Kaukinen et al., 2016; Neogi et al., 2016) but there are also conflicting results (Petersen et al., 2016). The histopathological scoring of synovitis in synovium obtained from OA patients during total knee arthroplasty showed a significant correlation between synovitis and pain intensity (Eitner et al., 2017). Further details on the relationship between subchondral bone features, pain and structural pathology in OA were reported in a recent comprehensive review (Barr et al., 2015). An intriguing question is which inflammatory mechanisms and mediators are mainly involved in pain generation. This issue will be addressed below.

### Animal Models of OA in Pain Research

In order to investigate pain mechanisms in OA, different OA models are employed. In rats often the monoiodoacetate (MIA) model of OA is used. The injection of MIA inhibits glycolysis and is toxic for chondrocytes. The pathology develops very rapidly. One day after MIA injection into the joint chondrocytes are shrunken and show fragmented pyknotic nuclei, the synovial membrane is expanded by fibrin proteinaceous edema fluid, and the joint is mildly infiltrated by lymphocytes, macrophages and plasma cells. Some days later, the inflammatory response in synovium subsides, necrotic cartilage collapses and chondrocytes are lost. Osteoclastic activity is increased, the subchondral bone collapses, and fragmentation of bony trabeculae surrounded by osteoclasts and some replacement by fibrous tissue and newly laid trabecular bone appear (for a review, see Schaible, 2013). The rapidly developing MIA model is clearly different from slowly developing human OA, and it displays in gene arrays substantial differences from human OA (Barve et al., 2007). However, it is considered useful to study pain mechanisms because it generates long-lasting mechanical hyperalgesia. Evidence for the presence of ongoing pain in the MIA model, assessed by conditioned place preference (CPP), was also reported (Liu et al., 2011).

In addition, or alternatively, models of joint destabilization are used in dog, sheep, rabbits, and guinea pigs, and in particular for pain research also in rodents. OA is induced by transection of anterior cruciate ligament, lateral meniscectomy, partial meniscectomy, menisceal tear, or combination of these measures (Bove et al., 2006). In surgically induced experimental OA, the first signs of hyperalgesia may appear only after a few weeks. Animals usually develop asymmetries in weight bearing and mechanical allodynia (a withdrawal response to a mechanical innocuous stimulus) upon paw stimulation with von Frey hairs, whereas testing for thermal hyperalgesia at the paw may yield inconsistent results (Fernihough et al., 2004; Bove et al., 2006; Malfait et al., 2010; Ferland et al., 2011; Mapp et al., 2013; de Melo Leite et al., 2014).

## Features of OA Pain and Neuronal Mechanisms of OA Pain

### Clinical Appearance of OA Pain

Initially, pain in OA joints occurs episodically during movements and loading of the joint (hyperalgesia), and this pain may be evoked by specific activities (Felson, 2009; Malfait and Schnitzer, 2013). These are typical features of nociceptive pain. At later stages, constant pain may occur, even at rest and at night (Felson, 2009; Ordeberg, 2009; Malfait and Schnitzer, 2013). Thus, there seems to be a gradual increase of pain with OA progression. However, it is unclear at which time point or at which stage of the OA process the pain starts.

**Figure 2:**
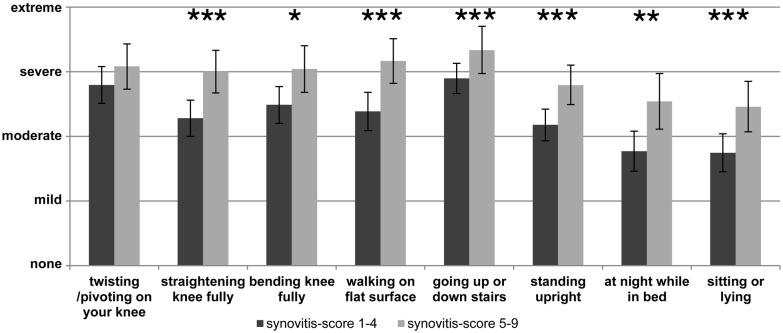
Assessment of pain at the knee with the Knee injury and Osteoarthritis Outcome score (KOOS) at the end stage of knee OA. The graph shows the severity of pain in patients with lower synovitis scores (1–4) and in patients with higher synovitis scores (5–9). Synovitis was scored using the histopathological scoring system of Krenn et al. (2005). It quantifies in points the enlargement of the lining cell layer (0: only 1 cell layer; 1: 2–3 cell layers; 2: 4–5 cell layers; 3: more than 5 cell layers), the cellular density of synovial stroma and pannus formation (0: normal cell density; 1: cell density slightly enhanced; 2: cell density moderately enhanced; 3: high cell density, multinuclear giant cells, pannus tissue) and leukocytic infiltrate (0: no infiltration; 1: single lymphocytes or plasma cells; 2: aggregates of lymphocytes; 3: dense infiltration with lymphocytes or lymph follicles). The total score ranges from 0 to 9 (0–1: no synovitis; 2–4: low-grade synovitis; 5–9: high-grade synovitis). The pain intensity had to be classified as either “none”, “mild”, “moderate”, “severe” or “extreme” (*n* = 70, mean ± 95% confidence interval, Mann-Whitney *U* test, *P < 0.05, **P < 0.01, ***P < 0.005). Reproduced from Eitner et al. (2017) with permission.

In the clinical setting pain and other symptoms of OA are usually assessed by questionnaires such as WOMAC (Carlesso et al., 2017) and/or in the case of knee OA by the “Knee injury and Osteoarthritis Outcome score” (“KOOS”) which is particularly used to document symptoms of the knee joint (Roos and Lohmander, 2003). An example of such a pain assessment with the KOOS pain score at the end stage of OA is displayed in Figure 2 which shows the responses to the individual questions. In Figure 2 two groups of OA patients are compared, namely patients with low synovitis scores and patients with higher synovitis scores (data from Eitner et al., 2017, for details see legend of Figure 2). The data show that pain at the OA knee joint occurs during normal activities which put load on the joint, and also under resting conditions, and they show that patients with a higher synovitis score suffer from more severe pain. These symptoms are an expression of a highly sensitized nociceptive system of the joint. A systematic review and meta-analysis revealed the presence of pain sensitization in knee OA patients (Fingleton et al., 2015). In particular the load-dependent pain components may be interpreted as features of nociceptive pain. Whether some of the symptoms, e.g., ongoing pain and night pain, indicate a neuropathic pain component is unclear (see below).

**Figure 3:**
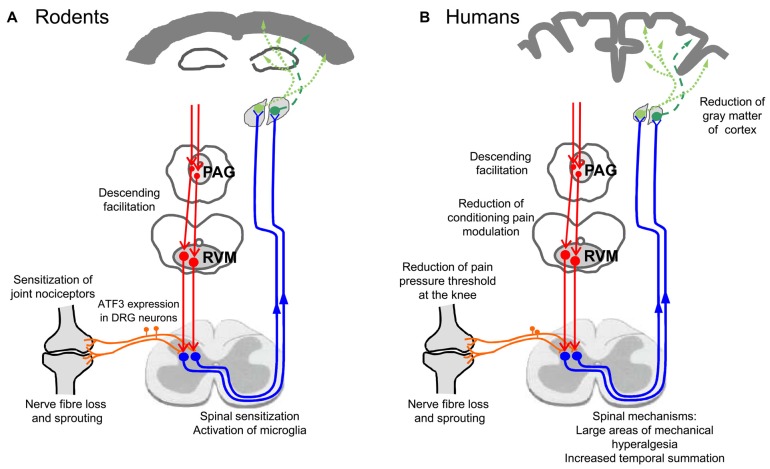
Neuronal changes in animal models of OA and in humans with OA. **(A)** Findings from studies on experimental OA models in rodents. **(B)** Findings in humans suffering from OA pain.

The active assessment of hyperalgesia of OA patients with a battery of tests was able to provide some insights into the neuronal mechanisms which are involved in OA pain. Many of these data show convergent results with electrophysiological studies obtained from experimental models of joint inflammation and OA, and therefore they will be presented side by side in the next paragraphs. To date, neuronal changes at several levels were identified to be at work in OA: peripheral sensitization, central sensitization, reduced descending inhibition, and atrophy of cortical areas. These changes may include neuropathic pain mechanisms and structural changes of innervation in the joint. Figure 3 shows an overview of mechanisms involved. Figure 3A summarizes findings from animal studies, Figure 3B depicts observations made in human studies on OA patients. These mechanisms are addressed in more detail below.

There is an ongoing discussion whether joint pain and in particular OA pain is in part neuropathic (Soni et al., 2013). According to the definition, neuropathic pain is a direct consequence of a lesion or disease affecting the somatosensory system, and there should be a plausible neuroanatomical distribution of the pain corresponding to a peripheral innervation territory (Treede et al., 2008; Thakur et al., 2014). Thus a neuropathic component would imply that the neurons innervating the joint undergo some damage, and such a condition may cause pain by itself, independently of the pathological changes in the joint. This could be important because the pathophysiology and the treatment of nociceptive pain and neuropathic pain are different (Schaible and Richter, 2004). Based on questionnaires such as PainDETECT and the description of pain by the patients, some reports and review articles concluded that a proportion of OA patients suffer from neuropathic pain. E.g., Hochman et al. (2010) reported that 34% of the patients in their study suffered from neuropathic pain because the patients complained about burning pain, tingling, numbness, more referred pain and high pain intensity. Dimitroulas et al. (2014) summarized some studies which reported neuropathic features of pain based on questionnaires. Still final conclusions are difficult to make. Not much information is available on nerve damage in OA in patients. Because the stimuli of quantitative sensory testing (QST) are mainly assessing cutaneous sensation, sensory changes or abnormalities in deep structures cannot be easily diagnosed with current QST (Thakur et al., 2014). Notably, central sensitization does not specifically indicate neuropathic pain (see below). However, the fact that some patients suffer from a loss of proprioception may indicate that complex somatosensory changes may take place which could include damage of neurons (Malfait and Schnitzer, 2013).

### Peripheral Neuronal Changes in OA

Joints are densely innervated by nociceptive A∂- and C-fibers, but under normal circumstances pain in a joint is only elicited by strong mechanical stimuli such as rotation against the resistance of the tissue and strong pressure. Noxious mechanical stimuli to the normal joint are encoded either by A∂- and C-fibers which have a high threshold (these fibers are selectively activated by frankly noxious stimuli such as overrotation and strong pressure) or by A∂- and C-fibers which show a small response to movements to the limit of the normal movement range and much stronger responses upon noxious stimulus intensities (Schaible and Grubb, 1993). A typical symptom of joint disease is the experience of pain during normal movements (see KOOS in Figure 2) and palpation, i.e., the threshold for the elicitation of pain by mechanical stimuli is lowered into the physiological range. This pain symptom is called mechanical hyperalgesia, and the basis for mechanical hyperalgesia is the sensitization of the nociceptive system to mechanical stimuli (Schaible and Grubb, 1993). Many of the patients with knee OA showed lowered pressure pain thresholds at the knee, at least in some areas (Arendt-Nielsen et al., 2014; Egsgaard et al., 2015; Neogi et al., 2015), a typical phenomenon of peripheral sensitization (Schaible et al., 2009). Signs of enhanced pain sensitivity upon application of mechanical stimuli were also seen in the DMM and MNX models (see above).

#### Sensitization of Joint Nociceptors

Experimental studies in anesthetized animals which recorded the activity of joint nociceptors and stimulated the exposed knee joint have shown that high threshold A∂- and C-fibers of the joint can be substantially sensitized for mechanical stimuli applied to the joint (mechanical sensitization). A long-lasting sensitization (hours, days) of mechanosensitive A∂- and C-fibers is observed during experimental models of inflammation, and in addition, primarily mechanoinsensitive (silent) nociceptors begin to respond to mechanical stimuli (Schaible and Grubb, 1993). The reduction of the mechanical pain threshold at the knee of OA patients suggests that joint nociceptors are also sensitized in OA. This was explored in two models of OA.

In the MIA model of knee OA, joint afferents of the knee were found to be sensitized to mechanical stimuli, and the amount of sensitization was correlated with the MIA dose (Schuelert and McDougall, 2009). Another study reported increased low rate spontaneous firing of C-fibers and an increase of mechanosensitivity (lowering of mechanical threshold and increased firing rate upon suprathreshold mechanical stimulation) only in A∂- but not in C-fibers (Kelly et al., 2012). In old guinea pigs with spontaneous OA, joint afferents exhibited increased firing upon noxious movements, but overall, there was no correlation between joint nociception and articular damage (McDougall et al., 2009). These data indicate peripheral processes affecting the discharge pattern of A∂- and C-fibers and show a peripheral component of sensitization.

#### Neuropathic Changes

In addition to sensitization, the MIA model provided evidence for a neuropathic pain component. Neuropathic mechanisms were proposed by the demonstration of ATF-3 immunoreactivity in DRG neurons (a marker of nerve injury) at 8 and 14 days of MIA-induced OA (Ivanavicius et al., 2007), the upregulation of galanin and neuropeptide Y and downregulation of substance P and CGRP in DRG neurons (a pattern typical for neuropathy; Im et al., 2010; but see Aso et al., 2016; who found an upregulation of CGRP), and the gabapentin sensitivity of the hyperalgesia (Ivanavicius et al., 2007). Based on ATF-3 immunoreactivity in DRG neurons it was proposed that neuropathic components are mainly induced when high doses of MIA are used (Thakur et al., 2012). The DMM model in rat showed similar changes of neuropeptides in DRGs as the MIA model (Im et al., 2010). Whether similar changes exist in DRGs of OA patients is unknown.

An interesting question is at which site(s) of the peripheral nerve fibers neuropathic changes are induced. The changes in the DRG neurons may represent responses of the cell bodies to damaging stimuli to nerve fibers in the peripheral tissue or in the peripheral nerve. However, under pathological conditions macrophages may invade the DRGs. An invasion of macrophages into the DRGs is typically found after nerve lesion (Hu and McLachlan, 2002; Hu et al., 2007), but was also detected in inflammatory joint models (Inglis et al., 2007; Segond von Banchet et al., 2009; Massier et al., 2015; Su et al., 2015). Miller et al. (2012) observed an infiltration of macrophages into the DRGs in the slowly progressive mouse DMM model by the onset of pain behavior, and this invasion was dependent on the chemokine CC-chemokine ligand 2 (CCL2), also known as MCP-1. Thus invasion of macrophages into the DRGs may occur in experimental neuropathy, experimental joint inflammation, and experimental OA. Whether DRGs of OA patients are infiltrated by macrophages is unknown. However, the presence of macrophages *per se* does not indicate their functional role in the DRGs. Dependent on their mode of activation, macrophages may assume different phenotypes and dependent on their phenotype they may have different effects on neurons. If they exhibit the M1 phenotype induced by classical activation (e.g., induced by LPS and interferon-γ) and express iNOS they may damage neurons. If they are less aggressive, e.g., after TNF stimulation, they release cytokines and can stimulate neurons without destroying them (Massier et al., 2015). In this context it is important to note that sensory neurons express ion channels and receptors not only at the sensory endings in the tissue but also in the cell bodies located in the DRGs. Thus the phenotype of the invading macrophages must be determined before firm conclusions can be drawn about their role.

#### Structural Changes of Innervation in OA

Not much is known about structural changes of innervation in OA. In a mouse model of OA induced by intraarticular injection of collagenase, an almost complete disappearance of CGRP- and substance P-positive nerve fibers was found in structures which were severely damaged, such as the fat pad of the patella and the synovial tissues, and some staining of irregular plate-like structures, without any clear morphology of nerve fibers, for GAP-43 was observed (Buma et al., 1992). GAP-43 is a marker of regenerating nerve fibers (Jochmann et al., 2015). In the MIA model in rat a significant reduction of DRG neurons was found which were retrogradely labeled from the knee joint (Ferreira-Gomes et al., 2010; Aso et al., 2016). However, in the study of Aso et al. (2016), there was a concomitant upregulation of CGRP and TrkA (the receptor of nerve growth factor (NGF)) in the nerve fibers retrogradely labeled from the knee and the subchondral bone.

Eitner et al. (2013) investigated the innervation of the parapatellar region of human OA knee joints in material obtained during total knee arthroplasty. Synovial regions without inflammation exhibited a dense vascular and neuronal network consisting of CGRP containing sensory fibers and tyrosine hydroxylase-positive sympathetic nerve fibers. However, in synovium with inflammatory changes there was a significant decrease of nerve fibers in depth ranges close to the synovial lining layer whereas deeper regions were less affected. Thus inflammatory changes in the synovium of OA joints are associated with a massive destruction of the capillary and neuronal network which is present in normal synovium. A reduction of peptidergic nerve fiber density in the synovium was also observed in inflammatory arthritis models (Murakami et al., 2015) and in synovium of patients with rheumatoid arthritis (Mapp et al., 1990). However, the picture is incomplete, because it is unknown whether the innervation also changes in deeper layers and other structures. In mice with complete Freund’s adjuvant-induced arthritis “hot spots” were identified at the synovial meniscal interface in which sensory as well as sympathetic fibers showed sprouting resembling neuroma formation (Jimenez-Andrade and Mantyh, 2012). Whether similar hot spots exist in OA joints is unknown. In humans with high chondropathy score increased vascular penetration and nerve growth in the meniscus was shown and discussed as a potential source of pain in OA (Ashraf et al., 2011).

Another study on human tissue (Suri et al., 2007) reported that vascular channels can break the tidemark between the subchondral bone and the OA cartilage and that some of these channels may also contain sympathetic and sensory nerves. This was observed in the articular cartilage in both mild and severe OA. Perivascular and free nerve fibers, and nerve trunks were observed within the subchondral bone marrow and within the marrow cavities of osteophytes. It was concluded that vascularization and the associated innervation of articular cartilage may contribute to tibiofemoral pain in OA across a wide range of structural disease severity (Suri et al., 2007). An MRI study pointed out that many patients with cartilage damage are asymptomatic and concluded that cartilage tissue nociception may be a rare event (Kaukinen et al., 2016). Collectively, these data indicate structural changes in the innervation. However, the functional significance of such changes needs to be further explored. In particular it is unknown whether they are an expression of neuropathic processes and whether they cause pain.

Changes of joint innervation may be relevant for OA pain as well as for the disease process. In inflammatory models it was shown that peripheral sensory neurons as well as the sympathetic nervous system can modify the inflammatory process (Pongratz and Straub, 2013; Schaible and Straub, 2014). Whether neuronal pathways play a role in the OA process should be explored. Since sympathectomy reduced both inflammation and behavioral hyperalgesia in an inflammatory model (Ebbinghaus et al., 2012a), it is of interest that in humans with OA the use of beta-blockers was associated with lower prevalence of joint pain and lower opioid requirement (Valdes et al., 2017).

### Central Sensitization

Increased nociceptive input from the joint causes complex changes in the central nervous system, which are called central sensitization. In the state of central sensitization, nociceptive neurons at different levels of the neuraxis are hyperexcitable, and hence, the nociceptive processing is amplified. Central sensitization has spinal and supraspinal components.

In the course of joint inflammation, nociceptive spinal cord neurons with joint input develop a state of hyperexcitability consisting of enhanced responses to mechanical stimulation of the joint (in the innocuous and the noxious range) and lowering of the excitation threshold of high-threshold spinal cord neurons. Furthermore, the neurons begin to show increased responses to stimuli applied to regions adjacent to and remote from the joint, and the total receptive field can exhibit an enlargement. These changes are the neuronal basis of primary hyperalgesia (at the site of disease) and secondary hyperalgesia (in areas adjacent to and remote from the joint; for a review, see Schaible et al., 2009).

At least at advanced stages of OA, patients show signs of central sensitization. They often report widespread pain beyond the OA joint (Ordeberg, 2009) and exhibit lower pressure pain thresholds at the OA joint and in cutaneous and subcutaneous structures of the whole leg (for reviews, see Schaible, 2012, 2013; Suokas et al., 2012). Patients with strong local hyperalgesia at the OA knee joint exhibit, in addition, higher temporal pain summation scores upon repetitive stimulation of the OA knee, which is another indicator for central sensitization in severe OA (Arendt-Nielsen et al., 2010; Neogi et al., 2015). Experiments on rats with MIA-induced knee OA support these conclusions, because these rats exhibit enhanced spinal responses to mechanical stimulation of the skin at the paw (Rahman et al., 2009; Sagar et al., 2010).

The mechanisms of central sensitization can be very complex, and the central sensitization during OA, in particular, has not been extensively studied. In OA models (MIA and surgical), the spinal content of substance P and CGRP is enhanced (Ferland et al., 2011; but see Im et al., 2010). Both neuropeptides are released from sensitized nociceptors and facilitate the generation of spinal hyperexcitability (Schaible et al., 2009). Spinal cord isolated from control and MIA rats, showed increased release of glutamate, bradykinin and CGRP in the spinal cord of MIA rats, and this release was reduced by a TRPV1 receptor antagonist (Puttfarcken et al., 2010). Besides the induction of spinal hyperexcitability by input from the OA joint, the labeling with markers of neuropathic pain in the DRGs (e.g., ATF3) raise the possibility, that neuropathic mechanisms may contribute as well. Indeed, in both the MIA model (Orita et al., 2011a; Sagar et al., 2011) and the DMM model (Tran et al., 2017) the microglia is activated, in the MIA model within a few days, in the DMM models after about 8 weeks, thus reflecting the time course of OA and pain development. However, although microglia activation is a prominent feature of neuropathic pain, it does not *per se* prove the presence of a neuropathic process because it occurs in inflammatory as well as in neuropathic conditions (Clark et al., 2007). An activation of microglia can even be observed functionally within 1–2 h after application of cytokines to the intact spinal cord (König et al., 2016), and in such a short period the generation of a neuropathic process is unlikely.

Spinal sensitization is often counteracted by local and descending inhibitory mechanisms. Rats with MIA-induced OA exhibited increased levels of the endocannabinoids 2-arachidonoyl glycerol and anandamide. Since spinally applied antagonists at the CB1 and CB2 receptor increased the responses of spinal cord neurons to cutaneous stimulation, it was suggested that endocannabinoids provide tonic spinal inhibition during OA (Sagar et al., 2010).

### Reduction of Descending Inhibition

Descending pathways from the brainstem mediate inhibition and facilitation of nociceptive spinal cord neurons. While descending inhibition is mainly observed in inflammatory pain states, neuropathic pain is often characterized by descending facilitation (Vanegas and Schaible, 2004). One form of *descending inhibition*, namely conditioning pain modulation (CPM, previously called diffuse inhibitory noxious control, DNIC), is out of order in patients with severe OA pain, but importantly, it is restored under pain-free conditions after joint replacement (Kosek and Ordeberg, 2000; Graven-Nielsen et al., 2012), combined with a reduction of widespread mechanical hyperalgesia (Graven-Nielsen et al., 2012).

In addition, *descending facilitation* may contribute to OA pain. In MIA-induced knee OA, serotoninergic descending facilitation contributes to responses of spinal neurons to innocuous mechanical stimuli applied to the area of cutaneous hyperalgesia in the paw (Rahman et al., 2009). In particular in rats in which MIA OA was induced at a high dose of sodium iodoacetate (thus creating a neuropathic component, see above) the ensuing central sensitization was in part mediated by descending facilitation, and it was improved by duloxetine, a selective serotonin and norepinephrine reuptake inhibitor which acts as an antidepressant and is effective against neuropathic pain (Havelin et al., 2016). In hip OA patients, functional imaging showed increased activation of the periaqueductal gray (PAG) in the brain stem during cutaneous stimulation in referred pain areas, which was interpreted as involvement of PAG in central sensitization (Gwilym et al., 2009). These data indicate that the severity of OA pain is partly determined by a distortion of the balance between inhibitory and excitatory modulating descending systems.

### Brain Activation

Imaging studies in humans identified brain areas that are involved in the generation of joint pain. In a positron emission tomography (PET) study patients with osteoarthritic knees showed increased activity in the cingulate cortex, the thalamus and the amygdala upon their accustomed physical activities, and this increase of activity was higher than during the application of experimental thermal pain stimuli applied to the knee region in pain free intervals (Kulkarni et al., 2007). Interestingly, like other chronic pain patients, patients with chronic OA pain exhibit signs of atrophy in the thalamus (Gwilym et al., 2010) and the gray matter of pain-related cortical areas (Rodriguez-Raecke et al., 2009). The functional implication of these changes is not known. Notably, these signs of atrophy were reversible after arthroplasty, and thus they seem to be, rather, a consequence than a cause of chronic pain (Rodriguez-Raecke et al., 2009). At advanced states, depressive symptoms and sleep disorders may occur (Felson, 2009; Malfait and Schnitzer, 2013).

### Neuronal Phenotype of Individual OA Pain Patients

**Figure 4:**
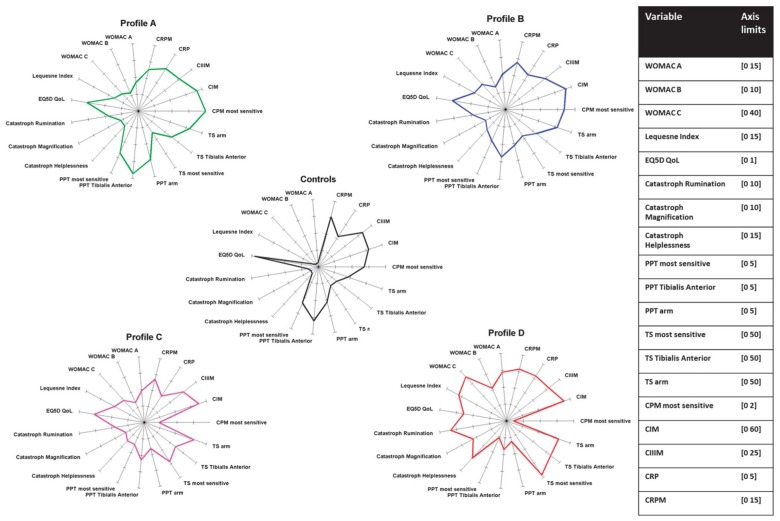
Neuronal phenotypes of pain in knee OA. The data are from Egsgaard et al. (2015) with permission. PPT, pressure pain threshold; TS, temporal summation; CPM, conditioning pain modulation; CIM, reflects MMP-mediated collagen type I; CIIIM, reflects MMP-mediated collagen type III (degeneration); CRP, C reactive protein; CRPM, MMP-mediated breakdown of CRP.

Figure 3 summarizes the neuronal alterations occurring in OA. Quite clearly, however, not all patients exhibit the same pain phenotype (Cruz-Almeida et al., 2013; Egsgaard et al., 2015; Frey-Law et al., 2016). According to Egsgaard et al. (2015) OA patients may exhibit different pain pattern most likely resulting from different combinations of neuronal mechanisms shown in Figure 3. They applied cluster analysis to 216 OA patients with joint pain and found four distinct knee pain profiles (Figure 4). Patients with knee pain profile A (27/216) had a higher pain threshold at the knee and a more efficient (CPM) than control persons, thus exhibiting low pain sensitivity and a highly efficient descending control. Patients with knee pain profile B (59/216) exhibited normal pain thresholds and normal CPM but exhibited enhanced temporal summation of pain during stimulation at some sites. Patients at this stage may be at a transitional stage from mild to moderate/severe pathology. Patients with knee pain profile C (85/216) showed low pressure pain threshold at the knee and other structures, enhanced temporal sensitization of pain for all stimulation sites, very low CPM, indicating full peripheral and central sensitization. These patients are considered the most typical OA patients. Patients of knee pain profile D (41/216) have similar changes as patients of profile C, and in addition they exhibit high scores in physical health questionnaires indicating strong sensitization and pain catastrophizing. Similar conclusions were also reported in a study on psychological profiles and pain characteristics of OA patients (Cruz-Almeida et al., 2013, see below). Another recent study used extensive QST measurements including tests of mechanical and thermal sensitivities and identified five sensitivity profiles (Frey-Law et al., 2016). Altogether these studies may provide a basis for a better understanding of neuronal mechanisms although it is unknown whether these pain sensitivities act as traits/enduring characteristics, or whether unique sensitivities develop subsequent to the onset of knee OA (Frey-Law et al., 2016).

## Mediators Involved in OA Pain

**Figure 5:**
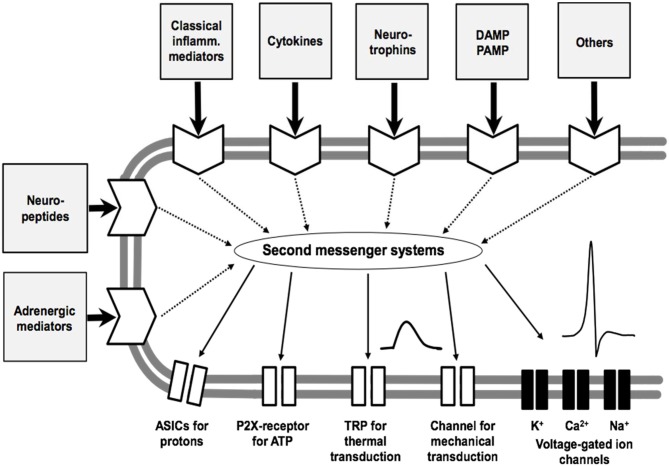
Model of the sensory ending of a nociceptive nerve fiber. The membrane at the bottom shows ion channels for transduction (which produce a sensor potential, SP), a voltage-gated Na^+^ channel for the generation of action potentials (APs), and voltage-gated K^+^ and Ca^2+^ channels that control excitability. The other part of the membrane displays receptors for mediators that act on different second messenger systems. Classical inflammatory mediators are bradykinin, prostaglandin E_2_, 5-hydroxytryptamine and histamine. ASIC, acid-sensing ion channel; P2X, purinergic ion channel; TRP, transient receptor potential; DAMP, damage-associated molecular pattern; PAMP, pathogen-associated molecular pattern. Modified from Schaible et al. (2011).

An intriguing question is whether key mediators can be identified in the joint which are particularly involved in the generation and maintenance of OA pain. Such mediators may be targets for pain treatment because they are key molecules in the process of sensitization of nociceptive sensory neurons. It is important to note, therefore, that most of the nociceptive sensory neurons are polymodal. They respond to noxious mechanical stimuli, proportions of them respond in addition to noxious thermal stimuli such as heat and cold, and they exhibit chemosensitivity for mediators. A simplified model of a sensory ending of a nociceptor is displayed in Figure 5. It expresses ion channels for the transduction of mechanical and thermal stimuli, voltage-gated Na^+^ channels for the generation of action potentials (APs), voltage-gated K^+^ and Ca^2+^ channels for the regulation of excitability, and ion channels and membrane receptors for numerous mediators, and receptors for damaging-associated molecular patterns (DAMPs) and pathogen-associated molecular pattern (PAMPS) such as Toll-like receptors (Schaible et al., 2011). The “chemosensitivity” for mediators has two purposes. Either a mediator such as H^+^ can directly open an ion channel (the ASIC, acid sensing ion channel) and thus evoke pain, or mediators bind to metabotropic membrane receptors which activate second messenger systems. The latter can influence ion channels of transduction and voltage-gated ion channels and thus change the sensitivity of the neuron for mechanical and thermal stimuli, and they change the excitability by influencing voltage-gated ion channels. Among the classical mediators are the prostaglandins and bradykinin. These mediators can cause a transient excitation and/or sensitization of the neurons. Of particular interest are mediators which cause a long-lasting sensitization for mechanical stimuli, and this group includes NGF and cytokines.

With respect to OA, the current situation is very complex for several reasons. Firstly, many mediators have been identified which are involved in the pathological process of OA. Only for some of them it has been tested whether they are directly relevant to pain by acting on nociceptive neurons. Secondly, many mediators were identified which may activate and/or sensitize joint nociceptors in experiments (for review, see Schaible, 2013) but it is unknown whether they are important in OA pain processes. Therefore, any list of mediators is incomplete. This review will mainly focus on NGF which binds to the TrkA receptor, and cytokines. NGF has been in the focus of pain research and pain therapy, cytokines are in the focus of both OA and OA pain research. Some data on the emerging role of chemokines in OA pain will be added.

### Nerve Growth Factor

NGF, a neurotrophin, is an essential growth factor for the development of normal nociceptors, and in the adult, a large proportion of nociceptors (expressing TrkA receptors, see Figure 5) remains dependent on NGF, which is required for their structural and functional integrity. Moreover, at inflammatory sites, several cell types produce NGF. *In vivo*, the application of NGF generates hyperalgesia. This is partly due to direct effects on neurons, and partly due to the stimulation of inflammatory cells by NGF to release inflammatory compounds. NGF enhances currents through TRPV1 channels (cation channels that are opened by heat and capsaicin), and reduces the threshold of thermal excitation. Long-term exposure to NGF increases the expression of TRPV1, bradykinin receptors, purinergic P2X receptors, and the synthesis of putative nociceptive transmitters such as substance P and CGRP in DRG neurons (Bennett, 2007). Thus, NGF is considered a key molecule for nociceptor biology, and it was shown to induce priming of nociceptors (Melemedjian et al., 2010). Priming means that after previous exposure to NGF or other compounds, the neuron is more responsive e.g., to prostaglandins. NGF can be produced by articular cartilage, meniscus, and synovium (Malfait and Schnitzer, 2013). In two experimental OA models the intraarticular injection of NGF augmented the behavioral pain responses (Ashraf et al., 2014).

In the mouse DMM model, NGF mRNA expression increased at day 3 and 16 weeks after the operation, but not in intervals that appeared pain free. A soluble NGF receptor, TrkAd5, effectively suppressed pain in both NGF-positive phases, whereas neutralization of TNF reduced pain only in the first phase, suggesting that OA-associated NGF may not be driven by classical inflammatory mediators (McNamee et al., 2010). Similarly, another study found in the MIA model a late increase of NGF (in conjunction with a neuropathic component), whereas in the first phase, TNF and IL-6 were elevated (Orita et al., 2011a). The anti-NGF antibody muMab911 prevented and reversed pain behavior and subchondral osteoclast numbers in the MIA model but did not alter cartilage and synovial pathology (Xu et al., 2016).

NGF became particularly interesting in recent years because in OA patients few injections of a monoclonal antibody against NGF provided persistent pain relief up to 56 weeks in a dose-dependent fashion and improved function in moderate to severe OA, with a low incidence of side effects (Lane et al., 2010; Nagashima et al., 2011; Schnitzer et al., 2011; Miller C. G. et al., 2015; Schnitzer and Marks, 2015). The initial studies were interrupted because some patients showed a worsening of OA associated with radiographic evidence of osteonecrosis and rapidly progressive OA during treatment, which required total joint replacements (Hochberg, 2015). Recently, however, allowance was given to restart clinical NGF antibody studies. Whether neutralization of NGF will become an option for OA therapy will depend on the effect on pain and the adverse effects (Hochberg, 2015; Miller C. G. et al., 2015).

### Cytokines

Proinflammatory cytokines such as IL-1β, IL-6, and TNF-α are not only key mediators in rheumatoid arthritis, they are also present in the joint in human and experimental OA (Bondeson et al., 2010; Im et al., 2010; Goldring and Otero, 2011; Orita et al., 2011a; Konttinen et al., 2012; Harkey et al., 2015; Larsson et al., 2015). They are mainly produced by chondrocytes, synoviocytes and macrophages. Chondrocyte-produced mediators are not carried away to the systemic circulation but access, via the interstitial tissue fluid and synovial fluid (SF), synovial lining cells and sublining tissues in particular (Konttinen et al., 2012). It is being discussed intensively whether cytokines are drivers of the OA process, in OA following trauma and in other forms of OA.

In general, it has been shown that proinflammatory cytokines can cause pain. First, they can induce the production of “classical pain mediators” such as prostaglandins which activate and/or sensitize nociceptive neurons. Second, cytokines can directly act on nociceptive sensory neurons many of which express receptors for cytokines such as TNF-α, IL-1β and others (Figure 5; for review, see Sommer and Kress, 2004; Schaible, 2014). Experimentally, a single injection of either TNF-α (Richter et al., 2010), IL-6 or of IL-6 and its soluble receptor sIL-6R (Brenn et al., 2007), IL-1β (Ebbinghaus et al., 2012b), or IL-17A (Richter et al., 2012) into the normal knee joint sensitized Aδ (TNF-α) and C-fibers (TNF-α, IL-6, IL-1β, IL-17A) to innocuous and noxious rotation of the knee joint at a slow time course (taking about 1 h to develop). The so-induced mechanical sensitization persisted for hours, which contrasts to the short-lasting mechanosensitivity increases after intraarterial injection of “classical mediators” (see above). In addition, cytokines are also involved in the central mechanisms of hyperalgesia. Cytokines in the central nervous system are either transported across the blood brain barrier, or they are produced in the central nervous system by microglial cells and to some extent by neurons (McMahon and Malcangio, 2009; Milligan and Watkins, 2009). In the MIA model significant increases of concentrations of IL-1α, IL-1β, TNF-α, IL-17, RANTES and other inflammatory mediators were found in the lumbar spinal cord (Im et al., 2010). However, the involvement of cytokines in pain in OA models was barely addressed. Yet, there are some indications from clinical studies that cytokines may be involved in the generation of OA pain, but so far the data are heterogeneous.

Orita et al. (2011b) found in knee OA with K/L grade 1–4 a positive correlation between the concentration of TNF-α in the SF and the pain intensity but no significant correlation between the TNF-α concentration and the radiographic grading. Notably the TNF-α concentration was smaller at later stages than at stage 1 (Orita et al., 2011b). Treatment of patients with knee OA, K/L grade 2/3, with the TNF-α antagonist adalimumab (6 subcutaneous injections of 40 mg adalimumab over 12 weeks) resulted in a significant improvement of WOMAC pain, stiffness and function as well as joint swelling (Maksymowych et al., 2012). In contrast, anti-TNF therapy with two subcutaneous injections of 40 mg adalimumab in patients with hand OA refractory to treatment with analgesics and NSAIDs failed to relieve pain (Chevalier et al., 2015). One injection of etanercept into the knee joint reduced the WOMAC scores of pain, stiffness and physical function at 4 weeks, and VAS was reduced at 1–2 weeks after injection (Ohtori et al., 2015). In erosive hand OA monthly intraarticular injection of infliximab reduced pain scores progressively (Fioravanti et al., 2009). Thus several studies showed pain reduction after TNF neutralization. It should be noted, however, that in patients with strong radiographic alterations or end-stage OA the concentration of TNF-α in SF may be very low or TNF-α may not detectable (Beekhuizen et al., 2013; see also Orita et al., 2011b) whereas higher TNF-α concentrations may be present at earlier stage of OA, particularly after previous injury. In the study of Beekhuizen et al. (2013) the concentration of IL-1β in the SF of patients with end-stage OA was also very low whereas the concentration of IL-6 in SF was elevated in early and end-stage OA.

Some clinical studies evaluated the effectiveness of the intraarticular injection of the IL-1 receptor antagonist anakinra or of the oral administration of the inhibitor diacerein on OA symptoms. Diacerein, a slow-acting agent, powerfully inhibits the IL-1β synthesis, reduces the IL-1R density, and inhibits the MEK/ERK intracellular cascades thus leading to a reduction of cytokines, NO, MMPs and ADAMTS (Martel-Pelletier and Pelletier, 2010). In patients with moderate to severe knee OA (K/L 2–3) the diacerein-groups (daily oral medication for 3–4 months) showed a significant reduction of VAS pain score and an improvement of WOMAC subscores compared with the placebo groups (Pelletier et al., 2000; Louthrenoo et al., 2007; Pavelka et al., 2007; Brahmachari et al., 2009; Kongtharvonskul et al., 2015). The positive diacerein effect outlasted the therapy at least 2 months (Louthrenoo et al., 2007; Pavelka et al., 2007). An intraarticular injection of 150 mg anakinra significantly reduced knee pain and improved function in patients with acute joint injury (Kraus et al., 2012) whereas in patients with knee OA a single injection of anakinra was not sufficient for a statistically significant improvement of WOMAC pain and function compared with placebo (Chevalier et al., 2009).

There is some indication of an association between the concentration of IL-6 in SF and the pain intensity of OA patients at various OA stages. The concentration of IL-6 was higher in patients with symptomatic knees compared with asymptomatic knees, and the patient-reported pain score was positively correlated with the IL-6 concentration (Cuellar et al., 2009). A correlation of elevated IL-6 and pain intensity was also found in patients with knee end-stage OA by Eitner et al. (2017). While several clinical trials showed an effective treatment of patients with rheumatoid arthritis with an anti-human IL-6 receptor antibody resulting in an improvement of symptoms of RA, similar studies on the inhibition of IL-6 signaling in OA patients were not found in the literature. In the database of clinical trials only one study is registered for the treatment of OA patients with the IL-6 receptor antagonist Tocilizumab. A double-blind, randomized, placebo-controlled trial was started in 2015 which evaluates the effect of Tocilizumab on pain relief in patients with severe hand OA (clinicaltrials.gov).

These data allow the following preliminary conclusions. First, different cytokines may be present at different stages of OA and thus it may be important to determine which cytokine is mainly present at a certain stage of OA. Second, it may be essential to treat for a sufficiently long time, and not to restrict the trial to patients who are unresponsive to other treatments.

### Chemokines

Since inflammatory processes came into the focus of OA research, chemokines (chemotactic cytokines) are of particular interest because they further synovial inflammation by stimulating leukocyte migration (Miller et al., 2014; Scanzello, 2017). Chemokines such as CCL2 (MCP-1), CCL3, CCL4, CCL5, CCL19, CCL21 are reported in the context of knee pathologies (trauma and OA), and notably, chemokine receptors such as CXCR2 and CXCR4 are also expressed in chondrocytes (Scanzello, 2017). For some chemokines (CCL2 = MCP-1, CX3CL1 = Fraktalkine, SDF-1) a positive correlation between the concentration in the SF and pain was reported in knee joint pathologies including OA in humans (Huo et al., 2015; Li and Jiang, 2015; Cuéllar et al., 2016). For CCL2 (MCP-1) it was shown that this chemokine sensitizes DRG neurons, and enhances Na_v_1.8 Na^+^ currents and the density of TRPV1 receptors (Miller et al., 2014). It was addressed above that MCP-1, which can be produced and upregulated in DRG neurons is involved in the invasion of macrophages into the DRGs (Miller et al., 2014). Thus research of the role of chemokines in OA pain deserves more attention.

## General Factors Which May Play A Role in OA Pain

OA is a disease which may result from numerous pathogenic factors (see the list of risk factors mentioned above and Figure 1). It is not the aim of this article to address the pathophysiological role of individual factors to the generation or progression of the disease OA. However, we believe that some of the risk factors may represent cofactors or comorbidities which have a direct or an indirect impact on the sensation of pain. According to common clinical experience, many patients with OA are elder persons, exhibit obesity, and suffer from a metabolic syndrome or diabetes mellitus (Berenbaum et al., 2017). This raises the question whether such comorbidities have a significant role in pain sensation (pain intensity, pain quality, localization of pain, etc.). So far this area of pain research is not well defined and therefore firm data are sparse.

### High Body Mass Index (BMI), Diabetes Mellitus

In many clinical studies on OA, numerous patients with OA exhibit obesity, i.e., a BMI >30 kg/m^2^. Apart from the fact that high body weight may put massive load on joints, obesity may be a significant comorbidity for a number of reasons. The adipose tissue may release proinflammatory cytokines (for review, see Berenbaum et al., 2017), create a low-grade systemic inflammation (Pitsavos et al., 2007; Berenbaum et al., 2017), and may be a risk factor for the development of the metabolic syndrome or diabetes mellitus (for review see Berenbaum et al., 2017). The metabolic syndrome is defined as the association of components that independently increase the risk of cardiovascular events, namely abdominal obesity, diabetes mellitus or insulin resistance, high cholesterol, and high blood pressure, and it is an independent risk factor for OA (Berenbaum et al., 2017). The abdominal adipose tissue of obese persons may contain high numbers of macrophages. The latter can produce cytokines such as IL-1β, TNF, IL-6, leptin, adiponectin and others (Wisse, 2004). Some proinflammatory cytokines regulate enzymes belonging to the metalloproteinase and ADAMTS families which are involved in cartilage degradation (Berenbaum et al., 2017). Also the fat pad in the knee joint is a source of proinflammatory cytokines (Ioan-Facsinay and Kloppenburg, 2013).

A frequent comorbidity of both obesity and OA is diabetes mellitus (Bays et al., 2007; Ganz et al., 2014). Diabetes mellitus may even be an independent risk factor for the development of severe OA (see above). In a 3-year follow-up study an increasing number of “metabolic risk factors” such as overweight, dyslipidaemia, impaired glucose tolerance, hypertension increased the risk of occurrence and progression of knee OA (ROAD study, Yoshimura et al., 2012). Diabetes mellitus may be an important pathogenic factor in OA for several reasons. First, diabetes mellitus may strongly influence the metabolism of cells, and it was actually implicated in dysfunctions of mitochondria (Sivitz and Yorek, 2010), the production of reactive oxygen species (ROS, Shenouda et al., 2011) and increased intracellular formation and leakage of methylglyoxal (MGO) which results in the formation of MGO-derived advanced glycation endproducts (AGEs, Maessen et al., 2015). It was proposed that such disturbances contribute to damage of chondrocytes (Mobasheri et al., 2002; Rosa et al., 2009) and an increased release of proinflammatory cytokines by synovial cells (Franke et al., 2009). Second, high glucose concentrations may increase the release of prostaglandin E_2_ (PGE_2_), IL-6, NO from chondrocytes after stimulation with IL-1β (Laiguillon et al., 2015). Third, diabetes mellitus may induce capillary dysfunction with negative effects on oxygen and glucose extraction (Østergaard et al., 2015).

### Effects of High BMI and Diabetes Mellitus on OA Pain

Diabetes mellitus was associated with increased hand pain in erosive OA (Magnusson et al., 2015), and aggravation of OA by diabetes mellitus may be reflected in enhanced OA pain (Schett et al., 2013). Eymard et al. (2015) reported an involvement of obesity but not of diabetes mellitus in pain. These data raised the question whether diabetes mellitus, or rather obesity, frequently associated with diabetic mellitus (Bays et al., 2007; Ganz et al., 2014), is an important factor determining the intensity of OA pain. Recently, we performed a study on knee OA patients and addressed the question whether the presence of obesity and diabetes mellitus influence pain intensity at the end stage of OA (Eitner et al., 2017). We compared pain intensity in non-diabetic patients with either normal body mass index (BMI) or with high BMI (median >30.1 kg/m^2^). The KOOS pain score of both groups were comparable, only standing upright was significantly more painful in patients with high BMI. By contrast, diabetic patients exhibited significantly increased pain intensity during loading the joint (fully straightening and bending the knee, walking on flat surface, going up- or downstairs, standing upright) and also during sitting and lying, and at night while in bed (Eitner et al., 2017). These data suggest that rather the diabetes mellitus and less the overweight causes enhanced OA pain. Additionally, patients with type 2 diabetes mellitus showed a worse functional outcome post total knee arthroplasty (Robertson et al., 2012), and a further study showed an association of diabetes with persistent pain after hip and knee replacement (Rajamäki et al., 2015).

An intriguing question is why and how diabetes mellitus enhances pain intensity. In the study of Eitner et al. (2017) diabetic patients exhibit more severe synovitis and higher concentrations of IL-6 in the SF than non-diabetic patients fitting to the finding that glucose enhances the release of cytokines from chondrocytes (Laiguillon et al., 2015). As addressed above, IL-6 can induce a long-lasting sensitization of joint nociceptors for mechanical stimuli, and is, therefore, a major cytokine to induce persistent mechanical hypersensitivity (Brenn et al., 2007). IL-6 “primes” nociceptors such that the sensitizing effect of PGE_2_ is prolonged and enhanced (Melemedjian et al., 2010). Another source of IL-6 and sIL-6R in the OA joint is the infrapatellar fat pad (Distel et al., 2009). MGO in the plasma, a highly reactive dicarbonyl byproduct of glycolysis and the major precursor in the formation of AGEs, enhances the excitability of DRG neurons and the firing of nociceptive neurons by acting on voltage-gated sodium channel Na_v_1.8 (Bierhaus et al., 2012). In mice, MGO facilitates neurosecretion of CGRP, increases COX-2 expression and evokes thermal and mechanical hyperalgesia (Bierhaus et al., 2012).

In this context it is important to note that diabetes mellitus may itself be a source of pain, in most cases by inducing a symmetrical sensory polyneuropathy in the distal part of the extremities, with prevalent loss of small fibers (Said, 2007; Callaghan et al., 2012; Scadding and Koltzenburg, 2013; Peltier et al., 2014). Severe diabetic sensory neuropathy may also cause painless foot ulcers (Scadding and Koltzenburg, 2013). Importantly, however, on average only about 10%–20% of the patients with diabetes mellitus develop painful neuropathy (Said, 2007; Callaghan et al., 2012; Scadding and Koltzenburg, 2013; Peltier et al., 2014).

### Other Factors with Effects on Pain

In addition to metabolic factors numerous other “general” factors may play a role in OA and OA pain. These factors can only be briefly addressed in this review.

#### Psychological and Socioeconomic Factors

As in all chronic pain conditions, psychological factors including pain catastrophizing were also found to be important in OA pain (Sale et al., 2008; Somers et al., 2009). In a study on 194 patients with knee OA psychological profiles and pain characteristics were assessed. Cluster 1 patients had high optimism with low negative affect, pain vigilance, anger, and depression along with the lowest self-reported pain/disability and the lowest sensitivity to mechanical, pressure, thermal pain. Patients of cluster 4, the other extreme, exhibited the highest pain vigilance, reactivity, negative effect, anger, and depression, and these patients were most sensitive to mechanical, pressure, and thermal stimuli, and showed significant central sensitization to mechanical and thermal stimuli (Cruz-Almeida et al., 2013). Individuals with knee OA who are at the highest risk of developing disability and pain are those who have a lower socioeconomic status (Cleveland et al., 2013).

#### Genetic Factors

From twin and other studies it became clear that there is a component of heritability in OA. Valdes and Spector (2011) concluded that OA is genetically heterogeneous with each individual common gene variant contributing only modestly to the risk of OA. Genetic influences play a role in various aspects of OA generation such as bone morphogenetic proteins, apoptosis and mitochondrial damage, extracellular matrix components, inflammation and immune responses, cartilage degrading enzymes, and pain (Valdes and Spector, 2011; Thakur et al., 2013). Warner and Valdes (2016) reviewed the main findings from analyses of genome-wide association scans, which assessed differences of ~400 genetic markers in the genome of patients with OA. Concerning OA pain, there are only few reports describing single nucleotide polymorphisms (SNPs) associated with pain, which implicated a genetic contribution to pain sensitivity. In one study, a genetic variant of catechol-O-methyltransferase (COMT) was associated with stronger hip OA pain (van Meurs et al., 2009). This association was not confirmed in cohorts with knee OA (Neogi et al., 2014). Another report described a variant in the TRPV1 gene. The *TRPV1* 585 Ile–Ile genotype was associated with a lower risk of symptomatic knee OA (Valdes et al., 2011b). Additionally, a SNP in the PCSK6 gene was strongly associated with protection against pain in knee OA (Malfait et al., 2012). Algesiometric assays revealed that PCSK6 knockout mice were also protected against pain (Malfait et al., 2012). Pain perception is also altered by a SNP in the SCN9A gene (encoding for the alpha subunit 9 of Na_v_1.7). Whereas Reimann et al. (2010) found an association of this genetic variant with higher OA pain scores, an analysis of three independent cohorts by Valdes et al. (2011a) confirmed only an involvement of this genetic variant in genetic susceptibility of pain but not in OA-specific pain. In three independent cohorts of patients with knee OA and non-OA controls, an increased interleukin-1 gene expression in peripheral blood leukocytes was associated with increased pain scores and predicts risk for progression of symptomatic knee OA (Attur et al., 2011). Recently, Warner et al. (2017) discovered a genetic variation at the neurokinin 1 receptor gene (TACR1) which was associated with pain in patients with knee OA. One of six tested SNPs in the TACR1 gene showed a nominally significant association with a decreased risk in symptomatic OA. This was replicated in four additional cohorts (Warner et al., 2017).

#### Innate Immunity

Miller R. E. et al. (2015) reported that DAMPs generated in murine OA directly excite murine nociceptive neurons through Toll-like receptor 4 (TLR4). This points to an involvement of innate immunity in OA pain mechanisms, showing an additional dimension of OA pain so far barely investigated.

## Considerations on Present and Future Therapeutical Possibilities

This final chapter will address some points which may be important for future work on OA, OA treatment and OA pain treatment. The first part will briefly address some question of monitoring of OA and OA pain. The second part will address some aspects of pain treatment with drugs.

### Monitoring of OA and OA Pain

The reported work suggests that there are marked differences between patients regarding the predominant pathophysiological mechanisms of OA and of the pain phenotype. It might be useful, therefore, to better analyze the pathological phenotype and the pain phenotype in order to define treatment options based on pathophysiological aspects of OA and pain in OA. Since any classification rests on available information there could be an important problem which was addressed by Dell’Isola et al. (2016). After the screening of the literature they proposed six subgroups of patients, namely patients in which chronic pain is the main problem, patients with strong inflammatory component, patients with metabolic syndrome, patients with the major problem on bone and cartilage, patients with mechanical overload, and patients with minimal joint disease. They believe that there might be considerable overlap of these “types” but this was difficult to work out because most of the literature addressed just one aspect. Thus a more comprehensive analysis is required taking into account all relevant aspects.

In order to obtain better analyses, methods beyond classical X-ray should be employed which allow to visualize inflammatory and other soft tissue changes. However, first it seems necessary to establish the methods which provide the best results. Concerning MRI, Riis et al. (2017) came to the conclusion that dynamic contrast-enhanced MRI, and here the parameter MExNvoxel (a surrogate of the volume and degree of synovitis) has the best correlation to histopathological synovitis. Concerning pain, pain biomarkers parameters such as local pressure pain thresholds at various sites, temporal summation of pain upon stimulation, CPM can be used to classify patients (Arendt-Nielsen and Petersen, 2017).

For arthroscopic surgery, near-infrared spectroscopy (NIRS) was developed to detect cartilage degeneration at an early stage. NIRS reflects changes in the cartilage composition, water-binding properties and mechanical features of cartilage (Spahn et al., 2008). NIRS can be used to distinguish between ICRS (International Cartilage Repair Society grading system) grade I lesions and healthy cartilage (Spahn et al., 2007).

For many reasons researchers look for soluble biomarkers which can be used for analysis. Soluble biomarkers might be used as a diagnostic tool to confirm the presence of OA, to determine the disease severity and to identify patients at an early stage of OA, as a prognostic tool to predict the progression of OA, or as a monitoring tool to control the treatment efficacy (Chevalier et al., 2017). This is also relevant for pain research because, as stated above, it is unknown at which stage of OA the disease becomes painful and because pain research is often confined to OA pain at the end stage. OA diagnosis by radiography, the so-called gold standard, is performed after clinical symptoms (e.g., pain) and loss of function have already appeared. But a treatment of OA with DMOADs (disease-modifying OA drugs) is necessary at an early stage and then more successful. Radiography is often inappropriate to evaluate clinical trials because radiographic changes are small over time (and may not be related to pain, see above). Therefore, the development of sensitive and predictive biomarkers is absolutely essential to optimize and monitor treatment interventions. Biomarkers are molecules or molecular fragments present in bone, cartilage or synovium and are related mostly to collagen metabolism, aggrecan metabolism, other non-collagenous proteins (such as cartilage oligomeric matrix protein: COMP, hyaluronic acid: HA, matrix metalloproteinase: MMP), or related to inflammatory processes, fibrosis and complement proteins (Lotz et al., 2013). An overview about biomarkers investigated for the evaluation of OA in presented by Lotz et al. (2013). There are a number of promising biomarkers but currently none of them is sufficient powerful for diagnosis, prognosis or control of clinical interventions. A reason for this may be that a biomarker concentration reflects a change in the metabolism of a tissue and is not necessarily related to the actual structural damage (Chevalier et al., 2017). But they can display disease-related biological processes in the joint. A combination of systemic biomarkers and imaging markers may have the power to detect early degeneration processes in the joint and to evaluate clinical trials. Currently, urinary C-terminal telopeptide of collagen type II (uCTX-II: a marker for type II collagen degradation) and serum COMP (a non-collagenous extracellular matrix protein present in cartilage) appear to be the most robust OA biomarkers. Most studies investigated the association of biomarkers with radiographic alterations.

Little is known whether the concentration of biomarkers is associated with pain. In patients with early radiographic knee OA uCTX-II, urinary N-terminal telopeptide of collagen type I (uNTX-I) and serum HA were significantly increased in patients with knee pain (Ishijima et al., 2011). Kraus et al. (2017) investigated 18 OA-related biomarkers of bone and cartilage turnover as predictor of knee OA progression over 4 years. Baseline levels of both uCTX-II and urinary alpha isomerized C-terminal telopeptide of collagen type I (uCTX-Iα) significantly predicted case status (subject cases that had both progressive pain and radiographic progression over a 4-year period). Baseline level of both uCTX-II and urinary Col2–3/4 C-terminal cleavage product of type II collagen human urine sandwich assay predicted pain worsening (Kraus et al., 2017). A meta-analysis of 32 studies including knee, hip and hand OA found that serum high sensitivity C reactive protein (hs-CRP**)** levels were significantly associated with OA pain and decreased physical function, whereas no significant associations were found between hs-CRP levels and radiographic OA (Jin et al., 2015). In a study on 67 patients with symptomatic knee OA the biomarker urinary glucosyl-galactosyl-pyrinoline (Glc-Gal-PYD) reflecting degradation of synovium was significantly associated with WOMAC pain and WOMAC total score and was the most important predictor of WOMAC index in this study (Garnero et al., 2001).

### Considerations on Treatment of OA and OA Pain

A recent review (Karsdal et al., 2016) summarized the current state of disease-modifying treatments for OA (DMOADs) of the knee and hip. It was stressed that OA is a heterogeneous disease of the whole joint which can be divided based on pathophysiological phenotype (bone-, cartilage-, or inflammation-focused) which may affect progression rates (see also above). It was concluded that the future treatment landscape in OA seems to contain several realistic possibilities with several structure-modifying drugs under development. This includes both systemic inhibitors of cartilage destruction such as ADAMTS inhibitors, and anabolics with the potential to regenerate cartilage in formulations such as sprifermin. Other candidates, such as antiresorptives specifically targeting the bone phenotype or anti-inflammatories such as anti-IL-1-α/β for the inflammatory phenotype are considered interesting candidates for selected and defined OA patient populations (Karsdal et al., 2016). This review did not, however, address pain as a direct target for treatment. One the other hand, recent developments in the field of pain research such as the use of antibodies to NGF do not address the role of NGF in the development of OA. Thus, research is still divided into the field of disease modification and the field of pain.

Current drug treatment for pain relief is mainly based on the use of acetaminophen (paracetamol), NSAIDs, opioids and glucocorticoids (Malfait and Schnitzer, 2013; Vergne-Salle and Beaulieu, 2017; Walsh, 2017). The use of duloxetine, a selective serotonin and norepinephrine reuptake inhibitor which acts as an antidepressant and is effective against neuropathic pain, was also found to be efficacious in the treatment of chronic OA pain (Citrome and Weiss-Citrome, 2012; Malfait and Schnitzer, 2013; Walsh, 2017). In the MIA model, duloxetine was mainly effective in rats which suffered from high dose MIA-induced OA, thus most likely exhibiting neuropathic pain (Havelin et al., 2016). However, the effect size of the paracetamol, NSAIDs and opioids is considered small to moderate, and inadequately controlled pain is the major reason for total joint replacement (Malfait and Schnitzer, 2013). The magnitude of pain relief by duloxetine was similar to that of NSAIDs offering an alternative to opioids for patients who are not able to take NSAIDs (Malfait and Schnitzer, 2013).

Because PGE_2_ is involved in peripheral as well as central sensitization (for review, see Telleria-Diaz et al., 2010; Natura et al., 2013), the use of cyclooxygenase inhibitors is rational. It should be noted, however, that different prostaglandins may have different functions, and that each prostaglandin may activate different receptor subtypes which may even produce opposite effects. For example, both PGE_2_ and PGD_2_ are involved in the sensitization of peripheral nociceptive neurons (Ebersberger et al., 2011; Natura et al., 2013) whereas in the spinal cord PGD_2_ may begin to counteract the nociceptive effect of PGE_2_ during peripheral inflammation (Telleria-Diaz et al., 2008). Furthermore, during inflammatory conditions, one of the EP receptors for PGE_2_, the EP3 receptor subtype, was shown to reduce peripheral and central sensitization thus counteracting the sensitizing EP2 and EP4 receptor subtypes (Natura et al., 2013). The antinociceptive EP3 receptor is strongly expressed in nerve bundles of synovial tissue of OA patients (Natura et al., 2013). Thus the inhibition of cyclooxygenases is not just antinociceptive. It may disturb the balance between sensitizing and desensitizing effects mediated by different prostaglandins and prostaglandin receptors. Whether agonists (such as an EP3 receptor agonist) or antagonists at specific PG receptors (antagonists at EP2 and EP4 receptors) are an option for pain therapy is unknown.

Opioids exert their antinociceptive actions in the peripheral and central nervous system. Peripheral opioid receptors were shown to produce profound antinociception experimentally as well as clinically (Stein et al., 2009). Whether peripherally acting opioids may become a therapeutical option is unclear.

The possible benefit of the neutralization of NGF was addressed above. An alternative may be the blockade of the TrkA receptor for NGF, because the TrkA inhibitor AR786 reduced pain and synovitis (but not osteochondral pathology) in the MIA and meniscal transsection model of OA (Nwosu et al., 2016).

The intra-articular injection of platelet-rich plasma (PRP) was developed to treat OA and cartilage injuries. PRP is prepared by centrifugation of autologous blood and includes a variety of growth and coagulation factors, adhesion molecules, cytokines, chemokines and integrins. Many of these substances can stimulate chondrocytes and multi-potent mesenchymal stem cells, induce synthesis of aggrecan and collagen type II, inhibit apoptosis and reduce the catabolic effects of inflammatory cytokines. PRP can release IL-1 receptor antagonist and soluble TNF receptor I and II, interferon γ as well as anti-inflammatory cytokines IL-4, IL-10 and IL-13 (Xie et al., 2014). These effects may result in a suppression of inflammation, protection of cartilage and reduction of OA pain. In a meta-analysis of 14 randomized controlled trials 12 studies favored PRP treatment compared with other injections including saline placebo, HA, ozone, and corticosteroids (Shen et al., 2017). However, another analysis of 15 randomized controlled trials mentioned methodological concerns and heterogeneity in the PRP protocols, thus concluding that definitive conclusions about the effects of PRP in OA patients are currently not possible (Bennell et al., 2017).

Other principles may become of interest. Approaches are either to target specific molecules in nociceptive neurons, such as specific ion channels, or to inhibit processes in the tissue which cause nociception. An example of the first approach is the use of sodium channel blockers. In the MIA model the intraarterial injection of A-803467, a blocker of the sodium channel Na_v_1.8 close to the knee joint, at day 14 of MIA, reduced the firing of joint afferents to noxious rotation (but not of innocuous rotation). Injection of A-803467 into the knee joint reduced the incapacitance and secondary hyperalgesia in MIA rats (Schuelert and McDougall, 2012). Na_v_1.8 is one of the sodium channels with a quite selective expression in nociceptive neurons and it is thought to contribute significantly to the generation of APs at the sensory endings (Waxman and Zamponi, 2014), and it is involved in the excitation of neurons by noxious mechanical stimuli (Dib-Hajj et al., 1998; Akopian et al., 1999; Abrahamsen et al., 2008). The currents through Na_v_1.8 are increased by PGE_2_ (England et al., 1996; Gold et al., 1998). In the MIA model an antinociceptive selective effect of lacosamide, an anticonvulsant that enhances the slow inactivation of voltage-gated sodium channels was only found in arthritic rats but not in control rats (Rahman and Dickenson, 2014). Furthermore, experimental data from the MIA model suggested the use of TRPV1 antagonists (Puttfarcken et al., 2010; Chu et al., 2011).

As an option for pain therapy, cannabinoids are discussed. Cannabinoids can act at CB1 and CB2 receptors. In the rat, CB1 receptor agonists cause antinociception in nociceptors of both the normal knee joint and MIA-induced OA knee joints, but the CB2 receptor agonist GW405833 reduced nociception in the normal knee joint but enhanced mechanonociception in OA knee joints (Schuelert et al., 2010). The enhanced nociception was attributed to an activation of TRPV1 in C- fibers, because this sensitizing effect was prevented by a TRPV1 antagonist. Thus, the effect of cannabinoids on mechanonociception in OA joints depends on whether CB1 or CB2 receptors are activated. In the MIA model CB2 receptors were upregulated in neurons and glial cells in the spinal cord, and the systemic application of the CB2 receptor agonist JWH133 reduced pain behavior, central sensitization and glial upregulation (Burston et al., 2013).

An approach targeting processes in the tissue is the *inhibition of osteoclast activity*. In the MIA model of rat pre-emptive chronic zoledronate treatment prevented pathological changes but in rats with advanced OA this treatment only partially restored bone mineral density and had a significant, but limited effect on pain (Strassle et al., 2010). In patients with knee OA a single infusion of zoledronic acid reduced VAS scores (but not the KOOS scores) of knee pain and reduced the area of bone marrow lesions within 6 months (Laslett et al., 2012). The use of osteoprotegerin reduced the development of pain behavior and joint pathology in the MIA model (Sagar et al., 2014). As mentioned above, the anti-NGF antibody muMab911 prevented and reversed pain behavior and subchondral osteoclast numbers in the MIA model but did not alter cartilage and synovial pathology (Xu et al., 2016).

## Conclusion

In order to improve the perspective of OA patients towards a life with less pain and restrictions, considerable efforts are necessary. The review tried to address the areas which are relevant for pain in OA patients: local processes in the joint, alterations of the nociceptive system, general factors such as comorbidities. The fact that OA is now in the focus of different disciplines will hopefully further a better understanding of OA pathology and the OA pain mechanisms and ultimately lead to better treatment options. The authors believe that research on OA pathology and OA pain would benefit from more integrative approaches to identify intersections between OA pathology and OA pain mechanisms. It remains to be determined whether mechanisms of OA pathology and of OA pain can be treated in a convergent manner or whether the disease and the pain remain entities which have to be addressed separately.

## Author Contributions

AE and H-GS revised literature, wrote the article, prepared figures. GOH revised literature, wrote the article.

## Conflict of Interest Statement

The authors declare that the research was conducted in the absence of any commercial or financial relationships that could be construed as a potential conflict of interest.
